# Association of Insulin Regimen and Estimated Body Fat Over Time among Youths and Young Adults with Type 1 Diabetes: The SEARCH for Diabetes in Youth Study

**DOI:** 10.1155/2022/1054042

**Published:** 2022-01-28

**Authors:** Anna R. Kahkoska, Angelica Cristello Sarteau, Daria Igudesman, Beth A. Reboussin, Dana Dabelea, Lawrence M. Dolan, Elizabeth Jensen, R. Paul Wadwa, Catherine Pihoker, Elizabeth J. Mayer-Davis

**Affiliations:** ^1^Department of Nutrition, University of North Carolina at Chapel Hill, Chapel Hill, NC, USA; ^2^Department of Biostatistics and Data Science, Wake Forest School of Medicine, Winston Salem, NC, USA; ^3^Lifecourse Epidemiology of Adiposity and Diabetes (LEAD) Center, University of Colorado Anschutz Medical Campus, Aurora, CO, USA; ^4^Department of Pediatrics, School of Medicine, University of Colorado, Aurora, CO, USA; ^5^Department of Pediatrics, University of Cincinnati College of Medicine, Cincinnati, OH, USA; ^6^Division of Endocrinology, Cincinnati Children's Hospital Medical Center, Cincinnati, OH, USA; ^7^Department of Epidemiology and Prevention, Wake Forest School of Medicine, Winston-Salem, NC, USA; ^8^Barbara Davis Center for Diabetes, University of Colorado School of Medicine, Aurora, CO, USA; ^9^Department of Pediatrics, University of Washington, Seattle, WA, USA; ^10^Department of Medicine, University of North Carolina at Chapel Hill, Chapel Hill, NC, USA

## Abstract

**Aims:**

To explore how changes in insulin regimen are associated with estimated adiposity over time among youths and young adults with type 1 diabetes and whether any associations differ according to sex.

**Materials and Methods:**

Longitudinal data were analyzed from youths and young adults with type 1 diabetes in the SEARCH for Diabetes in Youth study. Participants were classified according to insulin regimen categorized as exclusive pump (“pump only”), exclusive injections (“injections only”), injection-pump transition (“injections-pump”), or pump-injection transition (“pump-injections”) for each follow-up visit completed. Estimated body fat percentage (eBFP) was calculated using validated equations. Sex-specific, linear mixed effects models examined the relationship between the insulin regimen group and change in eBFP during follow-up, adjusted for baseline eBFP, baseline insulin regimen, time-varying insulin dose, sociodemographic factors, and baseline HbA1c (≥9.0% vs. <9.0%).

**Results:**

The final sample included 284 females and 304 males, of whom 80% were non-Hispanic white with mean diagnosis age of 12.7 ± 2.4 years. In fully adjusted models for females, exclusive pump use over the study duration was associated with significantly greater increases in eBFP compared to exclusive use of injections (difference in rate of change = 0.023% increase per month, 95%CI = 0.01, 0.04). Injection-to-pump transitions and pump-to-injection transitions were also associated with greater increases in eBFP compared to exclusive use of injections (difference in rate of change = 0.02%, 95%CI = 0.004, 0.03, and 0.02%; 95%CI = 0.0001, 0.04, respectively). There was no relationship between the insulin regimen and eBFP among males.

**Conclusions:**

Among females with type 1 diabetes, exclusive and partial pump use may have the unintended consequence of increasing adiposity over time compared to exclusive use of injections, independent of insulin dose.

## 1. Introduction

Youths with type 1 diabetes are exhibiting increasing rates of overweight and obesity according to data from US-based [1, 2] and international registries [3]. The cardiovascular health risk of excess adiposity, which may increase the risk of adverse events through increasing rates of dyslipidemia, hypertension, and insulin resistance, is of even greater concern among individuals with type 1 diabetes given the elevated cardiovascular disease risk associated with longstanding diabetes [4].

Weight management among youths with diabetes is notably complex because approaches for optimal weight status and glycemic control are inherently related and, at times, antagonistic [5–7], thereby yielding key clinical predictors of weight status that are unique to youths and young adults with type 1 diabetes. In particular, the intensive insulin therapy has been shown to induce weight gain [4]. Weight gain associated with intensive insulin therapy is likely multifactorial and relates to metabolic as well as behavioral factors [5, 8–10]. A key aspect of intensive insulin therapy is the mode of delivery, which may include daily injections or continuous subcutaneous insulin infusion (commonly referred to as insulin pump therapy). While there is a perception that, on an individual level, insulin pump therapy may be associated with weight gain [11], data on the long-term, population-level association between the insulin regimen and excess weight gain among youths and young adults with type 1 diabetes are limited with inconsistent findings [12, 13]. A further limitation is that data available from longitudinal studies of youths with type 1 diabetes are focused on weight status in terms of the body mass index (BMI) *z*-score [14], which does not directly reflect adiposity changes [15] and faces accuracy limitations when used to compare adiposity across sex, race, and stages of pubertal development [16].

The objective of the current analyses was to leverage a large, US-based nationally represented cohort to directly test how longitudinal patterns in the insulin regimen are associated with changes in adiposity over time and how this association differs according to sex.

## 2. Materials and Methods

### 2.1. Participants

The SEARCH for Diabetes in Youth study uses a population-based registry network at five sites in the United States to identify individuals diagnosed with any type of diabetes before twenty years of age [17]. The clinical sites include the state of South Carolina; Cincinnati, Ohio, and surrounding counties; the state of Colorado with southwestern US American Indian sites; Seattle, Washington, and surrounding counties; and Kaiser Permanente Southern California membership in seven counties, resulting in a catchment population of over 5.5 million children and adolescents aged <20 years [18]. Annual incidence of youth-onset diabetes in this population has been continuously ascertained since 2002 [18, 19]. Individuals diagnosed with type 1 or type 2 diabetes in 2002-2006 and 2008 were invited to participate in an observational research study on the natural history of youth-onset diabetes by completing baseline visits shortly after diagnosis (mean 9.6 (SD 6.4) months postdiagnosis) and, if completed, asked to return for visits at 12, 24, and 60 months as part of a longitudinal cohort study to measure risk factors for diabetes complications.

In 2011-2015 and 2015-2019, two additional, comprehensive follow-up “cohort” visits were conducted among those participants with ≥5-year diabetes duration for the assessment of health care quality, diabetes-related early complications, quality of life, and related characteristics. The study was approved by the institutional review boards with jurisdiction in each study location. All participants provided consent or assent as age-appropriate, and parents also provided consent for those aged <18 years.

The first cohort visit was completed by 2,777 participants at a mean age of 17.9 years (SD 4.8) and mean diabetes duration of 8.0 years (SD 2.0). The distribution of demographic, metabolic, and socioeconomic characteristics of participants who completed the first cohort visit was similar to that of the larger SEARCH registry population [20]. The second cohort visit was completed by 2,668 participants at a mean age of 21.5 years (SD 5.1) and mean diabetes duration of 11.2 years (SD 3.3). By design, approximately half of participants with type 1 diabetes who were non-Hispanic white were invited to complete the research visits for the cohort (target *n* ~ 700).

### 2.2. Data Collection

#### 2.2.1. Research Visits

Trained personnel administered questionnaires, conducted measurements of height, weight, and blood pressure, and obtained blood samples. The body mass index (BMI) was defined as weight (kilograms) divided by height (meters^2^) and converted to a *z*-score. A blood draw occurred after an 8-hour overnight fast, and medications, including short-acting insulin, were withheld the morning of the visit.

#### 2.2.2. Laboratory Measures

Blood samples were obtained under conditions of metabolic stability, defined as no episodes of diabetic ketoacidosis in the preceding month and the absence of fever and acute infections. The samples were processed locally and shipped within 24 hours to the central laboratory (Northwest Lipid Metabolism and Diabetes Research Laboratories, Seattle, WA). HbA1c was measured with a dedicated ion exchange high-performance liquid chromatography instrument (Tosoh Bioscience).

#### 2.2.3. Other Measures

Self-reported race and ethnicity were collected based on questions modeled after 2000 US Census and categorized as non-Hispanic white, non-Hispanic African American, Hispanic, and “other” (Asian-American, Native American, Asian Pacific Islander, other, and unknown). The use of self-report race/ethnicity has been addressed previously by the SEARCH study [21]. Health insurance type was classified as none, private, Medicaid, or other. Parental education was based on the highest educational level attained by either parent and classified as less than high school degree, high school graduate, some college through an associate degree, and bachelor's degree or more. Household structure was classified as two-parent household, single-parent household, or other structure. The insulin regimen was based on mode of insulin delivery (i.e., insulin pump, syringes, and insulin pen devices) and classified as insulin pump versus injections (including long-acting with rapid-acting insulin injections and 3 or more injections per day, long-acting with any other combination of insulin injections with 2 or more injections per day, any combination of insulins excluding long-acting insulin with 3 or more injections per day, and any combination of insulins excluding long-acting insulin with 2 injections per day or insulin once daily) [13]. The insulin dose was self-reported as a total daily dose standardized per kilogram of body weight. Frequency of self-blood glucose monitoring was self-reported and classified as <1 time per day, 1-3 times per day, and ≥ 4 times per day or use of continuous glucose monitoring (CGM). Physical activity and screen time were assessed using questionnaires. High physical activity was classified as self-reported vigorous activity 3–7 days weekly. Sedentary behavior was classified as 2 or more self-reported hours of screen time per day. Data from a validated food frequency questionnaire (FFQ) was available for 1,643 participants. Diet quality according to the 2010 Dietary Guidelines was assessed by the Healthy Eating Index (HEI) score, with a maximum value of 100 that signifies alignment with key dietary recommendations from the Dietary Guidelines for Americans [22].

### 2.3. Inclusion Criteria

For this analysis, we included participants with type 1 diabetes, defined based on the clinical diagnosis made by their physician or other health care provider collected from these clinicians or abstracted from medical records within 6 months of diagnosis and insulin use at follow-up. Participants were included if they were diagnosed at an age of 10 years or older, attended the baseline and at least two follow-up visits, and had complete data on insulin dose, insulin regimen, and body fat. Participants with a diabetes duration of less than 3 months at baseline were excluded to avoid weight change related to the diagnosis and initial treatment of T1D [23].

#### 2.3.1. Outcome Definition: Estimated Body Fat Percentage (eBFP)

Validated equations developed from 1999-2006 NHANES to predict percent body fat measurement in Americans 8 years and older [23] were used to generate a new eBFP variable. This variable was validated with DXA measurements in a nationally representative sample of youths. Equations incorporate age, race, weight, height, and waist circumference. Equations are sex- and race/ethnicity-specific (white, black, Mexican-American, and “other”), and participants who identified as Hispanic/Mexican American were modeled with the Mexican American equation; all others who did not fall into one of the three specific race/ethnicity categories were modeled with the “other” equation [23]. SEARCH previously operationalized these equations for the prediction of eBFP to examine longitudinal patterns of adiposity over time among youths with T1D [24].

#### 2.3.2. Exposure Definitions: Longitudinal Insulin Regimen Group

To avoid capturing early changes in regimens during the partial remission or “honeymoon” period that often occurs following diagnosis, we defined the longitudinal insulin regimen group beginning with the first follow-up visit. The baseline insulin regimen was included as a separate covariate in the models. Participants were assigned to one of four groups: injections only (i.e., using insulin injections at all follow-up visits), pump only (i.e., using an insulin pump at all follow-up visits), injections-pump (i.e., using injections and switching to a pump at a later follow-up visit), and pump-injections (i.e., using an insulin pump and switching to injections at a later follow-up visit). Four additional longitudinal insulin regimen patterns involving more than one change in the insulin regimen over the course of the follow-up were observed in the data but did not have sufficient sample size for analysis. This included injections-pump-injections (*n* = 13), pump-injections-pump (*n* = 9), injections-pump-injections-pump (*n* = 3), and pump-injections-pump-injections (*n* = 1). These groups were excluded from the analysis.

#### 2.3.3. Statistical Analyses

Demographic characteristics of individuals in the study sample were summarized using descriptive statistics. Given known sex-specific changes in body composition that occur during puberty, all analyses were stratified by sex. Sociodemographic and clinical characteristics were compared across sex using chi-squared tests for categorical variables and the *t*-tests for continuous variables. Change in eBFP from baseline to the last available follow-up visit was calculated for each participant, and participants were divided into quartiles of eBFP change. Quartiles were derived from the entire sample and then stratified by sex. We compared sociodemographic and clinical characteristics across quartiles of change in eBFP. Linear mixed effects models, stratified by sex, were constructed to evaluate associations between longitudinal insulin regimen groups and changes in eBFP over time. Using nonparametric smoothing approaches, it was determined that a linear model provided the best fit; therefore, we report results from linear models. Random intercepts and slopes were included. Interactions between the longitudinal insulin regimen and time (disease duration) were tested to examine whether changes over time in eBFP depended on the longitudinal insulin regimen. Models were adjusted for baseline eBFP, baseline insulin regimen, age at diagnosis, race/ethnicity, clinical site, baseline household income, baseline maximum parental education, baseline health insurance type, baseline HbA1c dichotomized as <9.0% or ≥9.0%), and insulin dose (included as a time-varying covariate). Finally, a three-way interaction between the longitudinal insulin regimen, duration, and insulin dose was tested to examine whether insulin group differences might vary by insulin dose. All *p* values were evaluated at the 0.05 significance level. Data analyses were performed using the statistical analysis software package SAS 9.4 (SAS Institute, Cary, NC).

## 3. Results

The study sample was comprised of 284 females and 304 males with type 1 diabetes. The relatively small sample size reflects participants that were excluded due to not having a baseline visit and at least two follow-up visits with complete data on the insulin regimen at those visits (*n* = 1,515). Baseline sociodemographic and clinical characteristics stratified by sex are depicted in Table 1. The sample was 80% non-Hispanic white with mean diagnosis age of 12.7 ± 2.4 years. Mean diabetes duration from baseline to the last follow-up visit was 124.2 ± 44.2 months (range 34.1-207.3m months for females) and 118.2 ± 40.3 months (range 34.1-214.9 months) for males. On average, the subgroup of males were older (*p* = 0.016), less likely to be a racial/ethnic minority (*p* < 0.001), and more likely to have private health insurance (*p* = 0.017) than the subgroup of females. Females scored significantly higher on the HEI (*p* = 0.001) and were less sedentary (*p* = 0.016) than males but had significantly higher eBFP (*p* < 0.001). The mean ± SD baseline eBFP was 32.4 ± 5.4% among females and 25.6 ± 5.8% among males (*p* < 0.001), with no significant differences in baseline BMIz. Females were also taking higher total daily insulin doses at baseline compared to males (*p* = 0.044).

### 3.1. Relationship of the Longitudinal Insulin Regimen and eBFP among Females

Baseline demographic and clinical characteristics according to quartile of change in eBFP from baseline to the last follow-up visit are shown for females in Table 2. Age at diagnosis decreased across quartile of eBFP change (*p* < 0.0001), while maximum parental education level increased (*p* = 0.024). Compared to other quartiles, the highest quartile of eBFP change contained the largest proportion of nonsmokers and the lowest proportions of former or current smokers (*p* = 0.003). Individuals in the highest quartile of eBFP change had the lowest baseline eBFP and BMIz score compared to other groups (*p* < 0.001). The baseline insulin regimen was not significantly different across quartiles of change in eBFP. There were no differences in eBFP quartile relative to baseline HbA1c, baseline daily insulin dose, and change in daily insulin dose.

Most females were in the injection-only (35.6%) and injection-pump (32.7%) longitudinal insulin regimen groups with fewer in the pump-only (22.2%) and pump-injection (9.5%) groups. In terms of the relationship to change in eBFP, there was a higher proportion of pump only (36.6%) and injections-pump (39.4%) in the highest quartile of change in eBFP relative to the other quartiles (overall *p* = 0.012).

**Figure 1:**
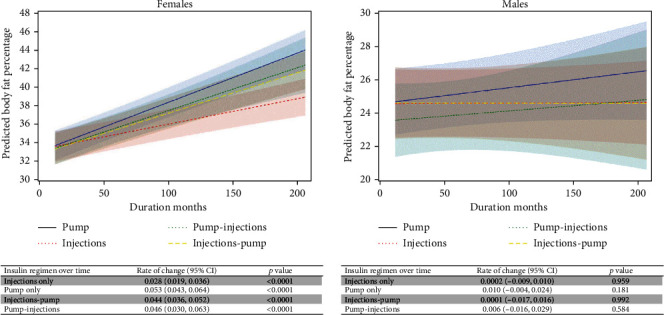
Adjusted, sex-specific trends in predicted body fat over time, stratified by insulin regimen. Models were adjusted for baseline predicted body fat percentage, baseline insulin regimen and baseline insulin dose, HbA1c, age at diagnosis, income, education, race, health insurance, and clinic site. Note that the *y*-axes are different for the female and male plots.

Following the baseline visit, in unadjusted models, eBFP increased on average 0.039% (95%CI = 0.034, 0.044) per duration month. As seen in Figure 1 and Table 3, eBFP increased significantly over time for each of the four insulin regimen groups in fully adjusted models. The estimated change in eBFP per duration month was greatest for pump only (0.053; 95%CI = 0.043, 0.064) followed by pump-injections (0.046, 95%CI = 0.030, 0.063), injections-pump (0.044, 95%CI = 0.036, 0.052), and injections only (0.028, 95%CI = 0.019, 0.036). As shown from the mixed model results in Table 3, injections only showed significantly smaller increases in eBFP than pump only (difference in rate of change of -0.026, 95%CI = −0.039, -0.012). Exclusive use of injections was also associated with smaller increases in eBFP compared to transitions from injections to pump use (difference in rate of change of -0.016, 95%CI = −0.028, -0.005) and transition from pump to injection use (difference in rate of change of -0.019, 95%CI = 0.038, -0.001). Change in eBFP was not significantly different between the other insulin groups, including pump only versus injection only, pump only versus pump-injections, and injections-pump versus pump-injections (Table 3). There was no interaction with insulin dose.

### 3.2. Relationship of the Insulin Regimen and eBFP among Males

Baseline demographic and clinical characteristics according to quartile of change in eBFP from baseline to the last follow-up visit are shown for males in Table 4. Age at diagnosis increased across quartile of eBFP change (*p* < 0.0001), while the proportion of nonsmokers decreased (*p* = 0.022). Physical activity levels were also different across groups, with quartiles 2 and 4 reporting the highest proportion of physically active individuals (*p* = 0.010). Baseline eBFP and BMIz decreased across quartile and were lowest in quartile 4 (*p* < 0.0001). Finally, there were significant differences in baseline insulin regimens, with higher use of insulin pumps or long-acting insulin with three or more injections per day in quartile 4 compared to other quartiles. Similar to females, there were no other differences in baseline HbA1c, baseline daily insulin dose, or change in daily insulin dose.

Most males were in the injection-only group (51.3%) followed by pump only (24.7%), injections-pump (16.1%), and pump-injections (7.9%). There were no differences in the proportions of injection-only, pump-injection, injection-pump, or pump-only users across quartiles of change in eBFP.

Following the baseline visit, in unadjusted models, overall eBFP did not change significantly (0.002; 95%CI = −0.004, 0.009; *p* = 0.46). As seen in Figure 1, there were also no significant changes in eBFP for any of the insulin regimen groups. As shown in Table 3, there were no significant differences in changes in eBFP over time between any of the insulin groups.

## 4. Discussion

In this study of 284 females and 304 males with type 1 diabetes, we found that the longitudinal insulin regimen pattern was associated with different trends in eBFP throughout youth and into young adulthood for females. By comparison, we found no associations between insulin regimen patterns and change in eBFP over time among males.

The present study contributes valuable quantitative insight to a still limited body of work examining intensification or change in the insulin regimen as clinical drivers of weight gain [9, 10]. While a series of analyses from the Diabetes Control and Complications Trial suggested that insulin intensification led to unintended weight gain [4, 25, 26], more recent analyses argue that this phenomenon is not necessarily true with current approaches. A recent report showed that a higher basal rate during pump therapy was associated with increased weight gain independent of total insulin dose [12]. However, other studies suggest that weight gain with transition from injections to pump therapy in adults does not inexorably lead to weight gain [27]: after one year, there was no significant change in weight reported from either of two clinical centers (Joslin Diabetes Center and Steno Diabetes Center). At both centers, modest weight gain (<2%) was observed among individuals with baseline HbA1c ≥ 9.0. However, the majority of currently available evidence comes from adults, and knowledge is limited about the relevance of this phenomenon in youth given the markedly different physiological processes that characterize puberty.

Intensification of insulin therapy has been examined in SEARCH previously, with analyses focused on change in HbA1c in relation to intensification of insulin regimens [28]. Although not a primary outcome, that study found that the BMI z-score was not significantly different between insulin regimen change groups at any time point, nor was BMI different by the insulin regimen and did not increase over time more dramatically on any particular regimen [28]. Herein, a more direct measure of excess adiposity compared to BMI and longer diabetes duration provided a critical tool to examine associations between the insulin regimen and adiposity in this large cohort setting. BMI conflates lean and fat mass, overestimates adiposity in males and underestimates adiposity in females, and inaccurately characterizes change in adiposity, particularly in children and adolescents [29–31]. The estimation equations that we used were developed from and validated in NHANES 1996-2006 using DEXA measurements, which is a reliable and valid method of monitoring body composition change in the growing youth [32].

Estimated body fat percentage has been operationalized previously to examine longitudinal patterns of adiposity over time among youths with type 1 diabetes and associations of trajectory group membership with sociodemographic factors [24]. The present analysis used a longitudinal, mixed modeling approach better suited to incorporate time-varying variables that may drive change in body composition over time. The overall trends in eBFP we found are consistent with previous reports showing increases in adiposity over time among females and decreases among males. The trends in eBFP are likely related at least in part to physiologic changes secondary to puberty [33]. This differential influence of puberty on eBFP change among males and females observed in our sample is in part illustrated by a pattern of decreasing mean age at diagnosis across increasing quantiles of eBFP change among females as compared to a pattern of increasing age across increasing quantiles of eBFP change among males. The change in eBFP is consistent with body fat percentile curves calculated using representative samples of American children and adolescents from NHANES, where body fat percentage peaks at approximately 11 years of age in boys before steadily decreasing and then leveling off by 15 years of age [34, 35].

A particularly striking finding of the present analysis is the sex-specific difference observed in the association between the insulin regimen and adiposity. The insulin regimen group was not associated with longitudinal trends in estimated adiposity among males. By contrast, among females, there was a statistically significant difference in estimated adiposity slopes between injections only versus pump only and injections only versus injections-pump in all models. When HbA1c was added as a covariate in the fully adjusted model, the difference in slopes between injection and pump-injection groups also became statistically significant. Interestingly, the association between the insulin regimen and body fat persisted with adjustment for insulin dose, and there was no interaction with time-varying total daily insulin dose, suggesting that patterns in the insulin regimen may serve as independent predictors of body fat over time. Given that the higher overall insulin doses that are potentially associated with more intensive regimens did not explain the association between insulin regimen intensification and gain in adiposity, there may be other drivers of weight change associated with partial and exclusive pump use, including different long-term patterns of exposure to basal and bolus doses across regimens.

Several other plausible behavioral and physiologic reasons may underlie our findings. More attention to self-management could influence parameters such as weight gain and glycemic control over time. There is anecdotal evidence that the use of an insulin pump is associated with stronger attention and adherence to self-management than the use of injections. However, it is also plausible that an injection regimen may be associated with more planning and consistency regarding timing and content of food intake (and less “grazing”) as opposed to perhaps greater flexibility of and variability in insulin and food patterns that may be more readily adopted on a pump regimen [36–38] and contribute to excess energy intake [39, 40]. Future studies that include more direct measures of diabetes self-management adherence will clarify the relationship between insulin regimen, adiposity, and specific characteristics of diabetes self-management behaviors that might shape the associations observed in the present analysis, including different eating patterns. Future studies are also needed to tease out whether plausible behavioral pathways, such as those posited above, are only applicable to females, in addition to shedding light on the physiologic differences that may make weight change vary by insulin regimen among females and not males during childhood and pubertal years. These studies may further reveal opportunities for noninsulin adjuvant therapeutics in the setting of type 1 diabetes to optimize weight status alongside glycemic control, particularly among subgroups predicted to show the greatest increase in body fat over time [41].

Future studies that include more direct measures of diabetes self-management adherence will clarify the relationship between insulin regimen, adiposity, and specific characteristics of diabetes self-management behaviors that might shape the associations observed in the present analysis, including different eating patterns and differences across sex. These studies may further reveal opportunities for noninsulin adjuvant therapeutics in the setting of type 1 diabetes to optimize weight status alongside glycemic control, particularly among subgroups predicted to show the greatest increase in body fat over time.

The study has several limitations. The small sample size warrants future larger studies to illuminate patterns that are both clinically and statistically significant. The equations to estimate eBFP have not been directly validated among youths with type 1 diabetes. Youths and young adults in the cohort spanned a broad age range at the same diabetes duration; thus, the study did not capture how the association between the insulin regimen and body fat may be different among youths diagnosed earlier in childhood versus later in childhood. Unfortunately, pubertal status was not evaluated at the time of the study visit for all participants, nor were variables that more directly capture diabetes self-management adherence and skills than the included socioeconomic variables. Data on other relevant clinical factors such as the presence of lipohypertrophy at the injection site or use of other medications were also not available. Although models were adjusted for age at diagnosis, heterogeneity in the pubertal stage across age may result in confounding by different physiologic or behavioral factors associated with the developmental stage. Data were only available from SEARCH study visits and therefore do not capture interim changes in the insulin regimen that may have occurred between visits, the inclusion of which could have resulted in grouping participants differently from the way they were categorized and compared in this study. There were a small number of youths who switched the insulin regimen more than once that were excluded from the analysis. Exclusion of these individuals may ultimately decrease generalizability to youths who may more frequently transition between insulin delivery modalities over time. Data on diet and physical activity were self-reported, with no corresponding objective measures.

Strengths of the study include the SEARCH for Diabetes in Youth cohort, which offers a longitudinal view including the transition from childhood into young adulthood. The eBFP variable offers a more specific measure of adiposity, elucidating changes over time as well as changes across sex that may be masked by BMI percentiles or *z*-scores.

In conclusion, data from a sample of a US-based and nationally representative longitudinal cohort of youths with type 1 diabetes advance current insight into type 1 diabetes-specific drivers of overweight and obesity by illuminating sex-specific differences in estimated longitudinal adiposity and their interactions with the insulin therapy regimen over time. Future studies that incorporate more specific measures of the pubertal stage and diabetes self-management and eating behaviors will help clarify the associations observed.

## Figures and Tables

**Table 1:** Characteristics of the SEARCH study participants at the baseline visit.

Data are reported as mean (SD) for continuous variables and *n* (%) for categorical variables.	All (*n* = 588)	Females (*n* = 284)	Males (*n* = 304)	*p* value
Demographics				
Age at diabetes diagnosis (years)	12.7 (2.4)	12.4 (2.4)	12.9 (2.5)	0.016
Age at baseline visit (years)	13.6 (2.4)	13.4 (2.4)	13.8 (2.5)	0.023
Race and ethnicity				
Non-Hispanic white	457 (77.7)	206 (72.5)	251 (82.6)	<0.001
Black	57 (9.7)	43 (15.1)	14 (4.6)	
Hispanic	60 (10.2)	31 (10.9)	29 (9.5)	
Other	14 (2.4)	4 (1.4)	10 (3.3)	
Health insurance type				
Private	475 (81.1)	217 (76.4)	258 (85.4)	0.017
Public	89 (15.2)	55 (19.4)	34 (11.3)	
Other/none	22 (3.8)	12 (4.2)	10 (3.3)	
Parental education				0.381
Less than high school graduate	20 (3.4)	9 (3.2)	11 (3.6)	
High school graduate	81 (13.8)	34 (12.0)	47 (15.5)	
Some college through an associate degree	197 (33.6)	104 (36.6)	93 (30.7)	
Bachelor's degree or more	289 (49.2)	137 (48.2)	152 (50.2)	
Health behaviors				
Smoking status				
Nonsmoker	496 (85.1)	240 (85.1)	256 (85.1)	0.902
Former	58 (9.9)	27 (9.6)	31 (10.3)	
Current smoker	29 (5.0)	15 (5.3)	14 (4.6)	
Healthy eating index (*n* = 537)	62.1 (6.9)	63.1 (7.0)	61.2 (6.6)	0.001
Physical activity				
0-2 days	218 (37.4)	115 (40.8)	103 (34.2)	0.102
3-7 days	365 (62.6)	167 (59.2)	198 (65.8)	
Time in sedentary behavior				
<2 hours per day of screen time	276 (47.5)	148 (52.7)	128 (42.7)	0.016
2+ hours per day of screen time	305 (52.5)	133 (47.3)	172 (57.3)	
Clinical characteristics				
eBFP (%)	28.8 (6.6)	32.4 (5.4)	25.6 (5.8)	<0.001
BMIz	0.53 (0.9)	0.51 (0.9)	0.54 (1.0)	0.740
BMIz category				
Underweight/normal (<85^th^ percentile)	389 (66.2)	188 (66.2)	201 (66.1)	0.523
Overweight (85^th^-<95^th^ percentile)	131 (22.3)	67 (23.6)	64 (21.1)	
Obese (≥95^th^ percentile)	68 (11.6)	29 (10.2)	39 (12.8)	
Insulin regimen				0.827
Pump	60 (10.2)	26 (9.2)	34 (11.2)	
Long-acting+short/rapid-acting insulin, 3×/day	203 (34.5)	98 (34.5)	105 (34.5)	
Long-acting+any other combination, 2×/day	44 (7.5)	24 (8.4)	20 (6.6)	
Any combo of insulins excluding long-acting, 3×	77 (13.1)	39 (13.7)	38 (12.5)	
Any insulin 1×day or combo excluding 2×	204 (34.7)	97 (34.2)	107 (35.2)	
Insulin total daily dose (units/kilogram)	0.71 (0.50)	0.75 (0.62)	0.67 (0.35)	0.044
HbA1c (*n* = 554)	7.65 (1.64)	7.76 (1.58)	7.55 (1.69)	0.135
HbA1c category (*n* = 554)				
HbA1c < 9%	458 (82.7)	211 (80.8)	247 (84.3)	0.283
HbA1c ≥ 9%	96 (17.3)	50 (19.2)	46 (15.7)	

eBFP: estimated body fat percentage; BMIz: body mass index *z*-score; HbA1c: hemoglobin A1c.

**Table 2:** Characteristics according to quartile of change in eBFP (baseline to last follow-up visit) for females.

Data are reported as mean (SD) for continuous variables and *n* (%) for categorical variables	Quartile 1 (<2.6%)	Quartile 2 (2.6% to 5.1%)	Quartile 3 (5.1% to 8.0%)	Quartile 4 (>8.0%)	*p* value
Demographics					
Age at diabetes diagnosis (years)	13.5 (2.5)	12.3 (2.5)	12.5 (2.3)	11.4 (1.7)	<0.001
Race and ethnicity^1^					0.127
NHW	42 (60.9)	58 (79.4)	54 (76.1)	52 (73.2)	
Black	15 (21.7)	6 (8.2)	10 (14.1)	12 (16.9)	
Hispanic	9 (13.0)	9 (12.3)	6 (8.4)	7 (9.9)	
Other	3 (4.4)	0 (0.0)	1 (1.4)	0 (0.0)	
Health insurance type^1^					0.060
Private	46 (66.7)	56 (76.7)	55 (77.5)	60 (84.5)	
Public	21 (30.4)	11 (15.1)	13 (18.3)	10 (14.1)	
Other/none	2 (2.9)	6 (8.2)	3 (4.2)	1 (1.4)	
Parental education^1^					0.024
Less than high school graduate	3 (4.4)	5 (6.8)	1 (1.4)	0 (0.0)	
High school graduate	14 (20.3)	9 (12.3)	9 (12.7)	2 (2.8)	
Some college through an associate degree	20 (29.0)	24 (32.9)	30 (42.2)	30 (42.2)	
Bachelor's degree or more	32 (46.4)	35 (48.0)	31 (43.7)	39 (54.9)	
Health behaviors					
Smoking status^1^					0.003
Nonsmoker	48 (69.6)	64 (88.9)	61 (87.1)	67 (94.4)	
Former	14 (20.3)	4 (5.6)	6 (8.6)	3 (4.2)	
Current smoker	7 (10.1)	4 (5.6)	3 (4.3)	1 (1.4)	
Healthy eating index (*n* = 263)	62.4 (6.6)	64.6 (7.3)	63.0 (7.4)	62.5 (6.8)	0.221
Physical activity					0.822
0-2 days	30 (43.5)	27 (37.5)	27 (38.6)	31 (43.7)	
3-7 days	39 (56.5)	45 (62.5)	43 (61.4)	40 (56.3)	
Time in sedentary behavior					0.614
<2 hours per day of screen time	36 (52.2)	39 (54.9)	40 (57.1)	33 (46.5)	
2+ hours per day of screen time	33 (47.8)	32 (45.1)	30 (42.9)	38 (53.5)	
Clinical characteristics					
eBFP (%)	35.6 (5.4)	32.6 (5.1)	32.5 (4.8)	28.9 (4.1)	<0.001
BMIz	0.96 (0.80)	0.58 (0.80)	0.56 (0.91)	-0.04 (0.95)	<0.001
Weight status					<0.001
Underweight/normal	31 (44.9)	49 (67.1)	49 (69.0)	59 (83.1)	
Overweight	27 (39.1)	17 (23.3)	13 (18.3)	10 (14.1)	
Obese	11 (15.9)	7 (9.6)	9 (12.7)	2 (2.8)	
Baseline insulin regimen					0.774
Pump	7 (10.1)	5 (6.8)	6 (8.4)	8 (11.3)	
Long-acting+short/rapid-acting insulin, 3×/day	23 (33.3)	24 (32.9)	27 (38.0)	24 (33.8)	
Long-acting+any other combination, 2×/day	9 (13.0)	6 (8.2)	3 (4.2)	6 (8.4)	
Any combo of insulins excluding long-acting, 3×	12 (17.4)	8 (11.0)	9 (12.7)	10 (14.1)	
Any insulin 1×/day or combo excluding 2×	18 (26.1)	30 (41.1)	26 (36.6)	23 (32.4)	
Longitudinal insulin regimen					0.012
Pump	12 (17.4)	12 (16.4)	13 (18.3)	26 (36.6)	
Injections	31 (44.9)	31 (42.5)	27 (38.0)	12 (16.9)	
Pump-to-injections transition	7 (10.1)	8 (11.0)	7 (9.9)	5 (7.0)	
Injections-to-pump transition	19 (27.5)	22 (30.1)	24 (33.8)	28 (39.4)	
Insulin total daily dose (units/kilogram)	0.75 (0.41)	0.75 (0.34)	0.83 (1.08)	0.66 (0.33)	0.486
Change in insulin daily dose	-0.004 (0.5)	0.08 (0.39)	-0.05 (1.0)	0.18 (0.48)	0.179
HbA1c (*n* = 260)	7.6 (1.7)	7.5 (1.3)	8.0 (1.7)	7.9 (1.6)	0.384
HbA1c category (*n* = 260)					0.445
HbA1c < 9%	50 (80.6)	56 (87.5)	54 (78.3)	51 (77.3)	
HbA1c ≥ 9%	12 (19.4)	8 (12.5)	15 (21.7)	15 (22.7)	

^1^Fisher's exact test with Monte Carlo standard error estimation. eBFP: estimated body fat percentage; BMIz: body mass index *z*-score; HbA1c: hemoglobin A1c.

**Table 3:** Adjusted differences in change in eBFP per diabetes duration month between insulin regimen groups stratified by sex.

Insulin regimen over time	Females	Males
	Difference in rate of change (95% CI), *p* value	Difference in rate of change (95% CI), *p* value
Injections only versus pump only	-0.026 (-0.039, -0.0125), <0.001	-0.009 (-0.026, 0.008), 0.281
Injections only versus injections-pump	-0.016 (-0.028, -0.005), 0.006	0.0003 (-0.019, 0.020), 0.973
Injections only versus pump-injections	-0.019 (-0.038, -0.001), 0.048	-0.006 (-0.031, 0.019), 0.627
Pump only versus injections-pump	0.009 (-0.004, 0.023), 0.159	0.010 (-0.012, 0.032), 0.381
Pump only versus pump-injections	0.007 (-0.013, 0.027), 0.488	0.003 (-0.024, 0.030), 0.812
Injections-pump versus pump-injections	-0.002 (-0.021, 0.016), 0.794	-0.006 (-0.035, 0.022), 0.653

Model adjusted for baseline predicted body fat percentage, baseline insulin regimen and baseline insulin dose, HbA1c, age at diagnosis, income, education, race, insurance, and clinic site.

**Table 4:** Characteristics according to quartile of change in eBFP (baseline to last follow-up visit) for males.

Data are reported as mean (SD) for continuous variables and *n* (%) for categorical variables	Quartile 1 (<2.6%)	Quartile 2 (2.6 to 5.1%)	Quartile 3 (5.1 to 8.0%)	Quartile 4 (>8.0%)	*p* value
Demographics					
Age at diabetes diagnosis (years)	11.4 (1.8)	11.8 (1.8)	13.7 (2.5)	14.8 (2.1)	<0.001
Race and ethnicity^1^					0.060
NHW	55 (72.4)	68 (86.1)	66 (88.0)	62 (83.8)	
Black	7 (9.2)	5 (6.3)	1 (1.3)	1 (1.4)	
Hispanic	10 (13.2)	4 (5.1)	8 (10.7)	7 (9.5)	
Other	4 (5.3)	2 (2.5)	0 (0.0)	4 (5.4)	
Health Insurance type^1^					0.063
Private	58 (76.3)	66 (83.5)	69 (92.0)	65 (90.3)	
Public	15 (19.7)	11 (13.9)	3 (4.0)	5 (6.9)	
Other/none	3 (4.0)	2 (2.5)	3 (4.0)	2 (2.8)	
Parental education^1^					0.259
Less than high school graduate	4 (5.3)	4 (5.1)	2 (2.7)	1 (1.4)	
High school graduate	13 (17.1)	9 (11.4)	14 (18.7)	11 (15.1)	
Some college through an associate degree	29 (38.2)	27 (34.2)	15 (20.0)	22 (30.1)	
Bachelor's degree or more	30 (39.5)	39 (49.4)	44 (58.7)	39 (53.4)	
Health behaviors					
Smoking status^1^					0.022
Nonsmoker	68 (90.7)	70 (89.7)	60 (80.0)	58 (79.4)	
Former	6 (8.0)	8 (10.3)	10 (13.3)	7 (9.6)	
Current smoker	1 (1.3)	0 (0.0)	5 (6.7)	8 (11.0)	
Healthy eating index (*n* = 273)	60.6 (6.4)	62.2 (7.1)	61.6 (6.4)	60.1 (6.4)	0.232
Physical activity					0.010
0-2 days	32 (42.7)	21 (26.9)	33 (44.0)	17 (23.3)	
3-7 days	43 (57.3)	57 (73.1)	42 (56.0)	56 (76.7)	
Time in sedentary behavior					0.840
<2 hours per day of screen time	30 (40.5)	34 (43.6)	30 (40.0)	34 (46.6)	
2+ hours per day of screen time	44 (59.5)	44 (56.4)	45 (60.0)	39 (53.4)	
Clinical characteristics					
eBFP (%)	30.9 (5.0)	25.7 (4.7)	24.1 (5.3)	21.4 (3.9)	<0.001
BMIz	1.15 (0.73)	0.37 (0.99)	0.51 (0.84)	0.12 (0.94)	<0.001
Weight status					<0.001
Underweight/normal	27 (35.5)	58 (73.4)	54 (72.0)	62 (83.8)	
Overweight	26 (34.2)	13 (16.5)	17 (22.7)	8 (10.8)	
Obese	23 (30.3)	8 (10.1)	4 (5.3)	4 (5.4)	
Baseline insulin regimen					0.002
Pump	7 (9.2)	9 (11.4)	8 (10.7)	10 (13.5)	
Long-acting+short/rapid-acting insulin, 3×/day	16 (21.1)	21 (26.6)	32 (42.7)	36 (48.6)	
Long-acting+any other combination, 2×/day	9 (11.8)	4 (5.1)	1 (1.3)	6 (8.1)	
Any combo of insulins excluding long-acting, 3×	12 (15.8)	9 (11.4)	7 (9.3)	10 (13.5)	
Any insulin 1×/day or combo excluding 2×	32 (42.1)	36 (45.6)	27 (36.0)	12 (16.2)	
Longitudinal insulin regimen					0.486
Pump	17 (22.4)	15 (19.0)	22 (29.3)	21 (28.4)	
Injections	36 (47.4)	44 (55.7)	37 (49.3)	39 (52.7)	
Pump-to-injections transition	10 (13.2)	4 (5.1)	6 (8.0)	4 (5.4)	
Injections-to-pump transition	13 (17.1)	16 (20.2)	10 (13.3)	10 (13.5)	
Insulin total daily dose (units/kilogram)	0.72 (0.38)	0.64 (0.35)	0.67 (0.32)	0.64 (0.36)	0.354
Change in insulin daily dose	0.08 (0.48)	0.40 (2.1)	0.15 (0.41)	0.20 (0.49)	0.357
HbA1c (*n* = 293)	7.5 (1.7)	7.5 (1.4)	7.6 (1.7)	7.6 (1.9)	0.972
HbA1c category (*n* = 293)					0.784
HbA1c < 9%	62 (86.1)	63 (84.0)	63 (86.3)	59 (80.8)	
HbA1c ≥ 9%	10 (13.9)	12 (16.0)	10 (13.7)	14 (19.2)	

^1^Fisher's exact test with Monte Carlo standard error estimation. eBFP: estimated body fat percentage; BMIz: body mass index *z*-score; HbA1c: hemoglobin A1c.

## Data Availability

Data are available on request from the authors.
